# Optimum DNA Extraction Methods for Edible Bird’s Nest Identification Using Simple Additive Weighting Technique

**DOI:** 10.3390/foods10051086

**Published:** 2021-05-14

**Authors:** Meei Chien Quek, Nyuk Ling Chin, Sheau Wei Tan

**Affiliations:** 1 Department of Process and Food Engineering, Faculty of Engineering, Universiti Putra Malaysia (UPM), Serdang 43400, Malaysia; qian_mc@hotmail.com; 2 Laboratory of Vaccines and Immunotherapeutics, Institute of Bioscience, Universiti Putra Malaysia (UPM), Serdang 43400, Malaysia; tansheau@upm.edu.my

**Keywords:** SDS method, Qiagen method, polymerase chain reaction (PCR), multiple attribute decision making (MADM) analysis, *Aerodramus*

## Abstract

A simple additive weighting (SAW) technique was used to determine and compare the overall performance of five DNA extraction methods from conventional (SDS method) to commercial kits (Qiagen, Wizard, and NucleoSpin) for identifying origins of edible bird’s nest (EBN) using end-point polymerase chain reaction (PCR). A hybrid method (SDS/Qiagen) which has been developed by combining the conventional SDS method with commercialised Qiagen was determined as the most suitable in terms of speed and cost-effectiveness. The determination of optimum extraction method was by the performances on efficiency and feasibility, extracted DNA concentration, purity, PCR amplifiability, handling time and safety of reagents used. The hybrid SDS/Qiagen method is less costly compared to the commercial kits and offered a more rapid alternative to the conventional SDS method with significant improvement in the yield, purity and PCR amplifiability. The developed hybrid SDS/Qiagen method provides a more practical alternative over the lengthy process using conventional method and expensive process using commercial kits. Using the simple additive weighting (SAW) technique and analysis, the Qiagen method is considered the most efficient and feasible method without consideration of cost as it yielded the purest extracted DNA and achieved the highest PCR amplifiability with the shortest turnaround time.

## 1. Introduction

Edible bird’s nest (EBN), also known as cubilose, is one of the most precious and expensive food produced from saliva of two swiftlet species, *Aerodramus fuciphagus* and *Aerodramus maximus* [1]. It is mainly originated from Southeast Asia countries, such as Indonesia, Thailand, Malaysia, and Vietnam [2]. Due to high demand and high price of genuine EBN, counterfeit and adulterated EBN are increasingly rampant in the markets. This has raised awareness of the importance of authentication of EBN. Several studies have employed DNA-based method to identify genuineness of EBN and its products [1,3,4]. The DNA-based method is known to be the most appropriate tool to identify species present in food [1] because DNA strands serve as templates for building new copies in cell replication, repair, and transcription. The DNA-based method is relatively faster, has greater sensitivity and specificity compared to the analytical and chemical methods when it comes to retrieving genetic information from food materials for species identification, varieties discrimination and allergy diagnosis [5,6,7].

Polymerase chain reaction (PCR) is a commonly used DNA-based method for identification and detection of food adulterant [8]. The mitochondrial cytochrome *b*, 12S rRNA and 16S rRNA genes are most widely used genetic markers for species identification by PCR due to availability of reference sequences in databases [9]. PCR method was developed to identify plant and insect origins of bee honey where markers of mitochondrial, nuclear, and chloroplast DNA were used to differentiate honey based on its origin [10]. A wide variety of meat products from different species like cattle, buffalo, sheep, goat, pig, chicken, ostrich, turkey, and rabbit were also authenticated by sequencing PCR products from a 555 bp region of mitochondrial cytochrome b gene [11]. Out of 20 commercial fresh and precooked products, 15% of them were found to be mislabeled. This method has also been applied for species identification of dairy products [12], fish [13] and meat [14], and detection of fruit ingredients in juices [15]. Despite being an accurate and efficient identification method, DNA-based method often faced challenges in terms of quality and quantity of extracted DNA which rely heavily on DNA extraction method. An efficient and reliable DNA extraction method must be effective in yielding adequate amount of high-quality extracted DNA and suitable for subsequent downstream molecular analyses such as conventional/end point PCR, real-time PCR, and DNA microarrays [16,17,18]. Various studies that have evaluated and compared DNA extraction methods on different subject matters are available [19,20,21,22].

As EBN naturally contains low amount of DNA, it is extremely challenging to extract good quality and sufficient quantity of DNA from EBN. The presence of abundant glycoprotein increases the difficulty to obtain high quality DNA [4]. The use of commercial kits are expensive while conventional methods are tedious, lengthy and hazardous. Lin et al. [4] employed two conventional methods, i.e., modified sodium dodecyl sulphate (SDS) and cetyltrimethylammonium bromide (CTAB) methods to overcome the two challenges in extracting DNA from EBN. Although the modified method can deliver good results, it was very time consuming and involved using sodium dodecyl sulphate reagent that can cause great hazard to human health. While existing DNA extraction protocols are available, they have not been compared comprehensively, specifically on EBN.

This work aimed to compare and determine the best method for efficient and feasible DNA extraction method for rapid species identification of EBN using a systematic analysis and engineering approach known as the simple additive weighting (SAW) technique. It is classified as a multiple attribute decision making (MADM) analysis. The hybrid SDS/Qiagen method, which is new, rapid, and cost-effective alternative was evaluated and compared with SDS method and three commercially available kits including Wizard Magnetic DNA purification system for food kit, NucleoSpin food kit, and DNeasy mericon food kit in terms of extracted DNA concentration, purity and PCR amplifiability, plus the time, cost, and safety of the extraction method. The optimal DNA extraction method for EBN was identified using simple additive weighting technique and validated for applicability for species identification of EBN through end-point PCR.

## 2. Materials and Methods

### 2.1. Edible Bird’s Nest Preparation

The 13 types of EBN samples originated from two swiftlet species, *A. fuciphagus* and *A. maximus* were collected from Malaysia (Table 1). The 11 unprocessed EBN samples were obtained directly from local farmers and two processed EBN samples were purchased from local markets. Processed EBNs have undergone harvesting, sorting, soaking, cleaning, moulding, drying, and packaging processes. The unprocessed EBNs were cleaned manually using tweezers to remove loose feathers and impurities. The EBN samples were then pulverised with liquid nitrogen using mortar and pestle, sieved through 1 mm mesh size to obtain a homogenous and fine powder for optimum yield. The samples were stored at 4 °C until DNA extraction. Two fake EBN samples were also used as samples and they were subsequently omitted in analysis due to negative results of extracted DNA.

### 2.2. DNA Extraction

Total genomic DNA of EBN samples were extracted using five different DNA extraction methods, namely Wizard (Promega Corporation, Madison, WI, USA), NucleoSpin (Macherey-Nagel GmbH and Co. KG, Düren, Germany), Qiagen (Qiagen Corporation, Hilden, Germany), SDS, and SDS/Qiagen. Each EBN was extracted in quadruplicate to ensure reproducibility of the extraction methods. For fair comparison of all extraction methods, the amount of starting materials was standardised to 25 mg of EBN samples and the final volume of extracts was fixed at 100 µL. The extracted DNA was stored at −20 °C. 

### 2.3. Wizard Magnetic DNA Purification System for Food Kit (Wizard Method)

The EBN samples were extracted using commercial kit, Wizard^®^ Magnetic DNA purification system for food (Promega Corporation, Madison, WI, USA) following the manufacturer’s instructions except the volume adjustments in lysis buffers. Each EBN of 25 mg was vigorously vortexed with 450 µL of Lysis Buffer A and 5 µL of RNase A, then vortexed again with 200 µL of Lysis Buffer B for 15 s in a 1.5 mL microcentrifuge tube. The tube was laid on its side and incubated at room temperature for 10 min. The sample was vigorously vortexed with 700 µL of precipitation solution and centrifuged at 13,000× *g* for 10 min in a 5415D microcentrifuge (Eppendorf, Hamburg, Germany) for protein precipitation. About 700 µL of supernatant was vortexed with 50 µL of resuspended MagneSil™ paramagnetic particles (PMP) in a new microcentrifuge tube, and then continued with remaining procedures in the manufacturer’s instructions. The Wizard method lyses with guanidine thiocyanate and RNase, and binds DNA to silica-coated magnetic beads.

### 2.4. NucleoSpin Food Kit (NucleoSpin Method)

NucleoSpin^®^ food kit (Macherey-Nagel GmbH and Co. KG, Düren, Germany) was performed with minor modifications by doubling the lysis buffers volume and prolonging the incubation time. About 25 mg of each EBN was mixed with 1100 µL of preheated Lysis Buffer CF at 65 °C and 20 µL of proteinase K. The sample was incubated at 65 °C for 1 h in a ThermoStat plus heating block (Eppendorf, Hamburg, Germany) and centrifuged at 11,000× *g* for 10 min to pellet contaminations and cell debris. Then, 450 µL of clear supernatant was vortexed with 450 µL of binding buffer C4 and 450 µL of 96% (*v*/*v*) ethanol, and followed with DNA binding, washing, and elution steps in the manufacturer’s instructions. The NucleoSpin method lyses with chaotropic salts, denaturants, detergents, and proteinase K, and binds DNA to silica membrane in spin column.

### 2.5. DNeasy Mericon Food Kit (Qiagen Method)

The Qiagen method was conducted using DNeasy^®^ mericon™ food kit (Qiagen GmbH, Hilden, Germany) following the manufacturer’s instructions with slight alterations. Each EBN of 25 mg was vortexed with increased volume of 1.3 mL food lysis buffer and 5 µL proteinase K, and then incubated for a longer period of 1 h at 60 °C to enhance inhibitor precipitation. The following extraction procedures were proceeded with the manufacturer’s instructions until the elution step, where DNA was eluted from QIAquick spin column with 100 µL of buffer EB instead of 150 µL for standardisation purpose. The Qiagen method lyses with non-ionic detergent CTAB and proteinase K, and binds DNA to silica membrane in spin column.

### 2.6. Conventional SDS Method (SDS Method)

SDS method was performed following Lin et al. [23] with some modifications. About 25 mg of each EBN was added with 1.2 mL of lysis buffer (10 g/L SDS, 50 mM Tris-HCl pH 8.0, 10 mM EDTA pH 8.0, 0.04 M DTT, 200 mg/L proteinase K, 2.0 M NaCl preheated at 65 °C) in a microcentrifuge tube. The mixture was vortexed and incubated at 65 °C for 1 h, followed by centrifugation at 12,000× *g* for 5 min at 4 °C to remove undigested debris. A 1000 µL of supernatant was transferred to a new tube containing equal volume of chloroform/isoamyl alcohol solution (24:1) and mixed well before centrifuged to remove protein. Supernatant of 500 µL was added with 50 µL of 10% CTAB/0.7 M NaCl buffer preheated at 65 °C and incubated at room temperature for 15 min, then mixed with 500 µL chloroform/isoamyl alcohol solution. The mixture was centrifuged to remove remaining CTAB and glycoprotein, and 400 µL of supernatant was transferred to new tube. The supernatant was mixed with 280 µL of cold isopropanol and centrifuged for DNA precipitation. The pellet was washed with 1 mL of 75% (*v*/*v*) ethanol and centrifuged at 12,000× *g* for 5 min. The DNA pellet was air dried and resuspended in 100 µL of nuclease-free water. The SDS method lyses with anionic detergent SDS, DTT and proteinase K, purifies DNA with cationic detergent CTAB and chloroform/isoamyl alcohol, and precipitates DNA with cold isopropanol.

### 2.7. Hybrid SDS Method and Qiagen Method (SDS/Qiagen Method)

Hybrid SDS/Qiagen method was developed by combining the SDS method and Qiagen method. The EBN samples were lysed using the SDS method, and then the following steps of DNA binding, washing, and elution were performed using QIAquick spin column from the Qiagen method. The initial procedure of this method was similar to the SDS method, from sample lysis to the addition of cold isopropanol steps. After mixing 400 µL of supernatant with 280 µL of cold isopropanol, the mixture was transferred to the spin column and then proceeded with remaining procedures in the Qiagen method. This SDS/Qiagen method lyses with anionic detergent SDS, DTT, and proteinase K, purifies DNA with cationic detergent CTAB and chloroform/isoamyl alcohol, and binds DNA to silica membrane in spin column.

### 2.8. DNA Quantification and Purity 

DNA concentration of EBN samples was quantified with spectrophotometric assay by measuring UV absorbance at 260 nm (A_260_) using a BioSpectrometer^®^ kinetic spectrophotometer (Eppendorf, Hamburg, Germany). One-way analysis of variance (ANOVA) was performed to compare the DNA concentration between five different DNA extraction methods (Table 1). Significant differences between means were evaluated using Tukey’s test at a confidence level of 95%. Fluorometric quantification assay was also performed based on fluorescent DNA binding dyes using a Qubit^®^ 2.0 fluorometer (Life Technologies, Carlsbad, CA, USA) and Qubit^®^ dsDNA high sensitivity assay kit. This assay is highly specific and selective for double-stranded DNA (dsDNA) quantification. Purity of the extracted DNA was determined by the absorbance ratios of 260 and 280 nm (A_260_/A_280_) using the spectrophotometer.

### 2.9. PCR Amplification

PCR amplification was performed to compare the performance of five different DNA extraction methods. The extracted DNA of EBN samples were amplified using mitochondrial cytochrome b gene primers available in the literature, L15302 (5′ GTA GGA TAT GTC CTN CCH TGA GG 3′) and H15709 (5′ GGC ATA TGC GAA TAR GAA RTA TCA 3′) to amplify 406 bp PCR products (S1) [24]. These primers were synthesised by AIT Biotech in Singapore. PCR amplification was conducted in a 50 µL total reaction volume containing final concentration of 1 x MyTaq™ Mix PCR buffer (Bioline, London, UK), 0.4 µM of each forward and reverse primer, and 0.02–1.70 ng/μL of DNA template. A mixture with no DNA template was used as negative control. The amplification was performed using a C1000 Touch™ thermal cycler (Bio-Rad, Hercules, CA, USA) with the following PCR cycle: initial denaturation at 95 °C for 3 min; followed by 35 cycles of denaturation at 95 °C for 15 s, primers annealing at 53 °C for 30 s and extension at 72 °C for 30 s; then final extension was conducted at 72 °C for 5 min. Each PCR amplification was performed in at least triplicate to ensure its repeatability.

PCR products were analysed by agarose gel electrophoresis using a 1.5% (*w*/*v*) agarose gel pre-stained with Red-Safe™ DNA dyes (iNtRON Biotechnology, Sungnam, Korea) at 80 V for 60 min. A 100 bp HyperLadder™ DNA ladder (Bioline, London, UK) was used as PCR products size marker. The gel was visualised under UV light using a Gel™ Doc XR imaging system (Bio-Rad, Hercules, CA, USA). The expected PCR product size was 406 bp. 

### 2.10. DNA Sequencing

The PCR products were sequenced using an ABI3730x1 automated DNA sequencer (Applied Biosystems, Foster City, CA, USA) and the same primers used in PCR amplification. The nucleotide sequences obtained were subjected to the nucleotide basic local alignment search tool (BLASTN) available at National Centre for Biotechnology Information (NCBI) (http://blast.ncbi.nlm.nih.gov/Blast.cgi (accessed on 25 May 2015)) for sequence similarity search. Generally, if PCR product sequence and database sequences show maximum identities or highest similarities, the identity of EBN samples can be confirmed.

### 2.11. Ranking of DNA Extraction Method Using Simple Additive Weighting Technique 

Multiple attributes including dsDNA concentration, purity, PCR amplifiability, procedure simplicity, safety of reagents (beneficial attributes), and handling time (non-beneficial attribute) were used to evaluate overall performance of DNA extraction method. Cost was evaluated based on a relative qualitative scale. As some of the attributes are contradictory, it increased the difficulty in selecting the optimal extraction method. A simple additive weighting (SAW) technique of multiple attribute decision making (MADM) analysis was used. This technique clustered all attributes results into a comprehensive system based on mathematical scoring technique thus provided ranking to each DNA extraction method [25]. All attributes used were normalised to standardised values to ensure they contribute evenly to a scale for comparison purposes [26]. The level of procedure simplicity and reagents safety attributes were rated based on direct rating method [27], where 1 and 2 indicated simple and more simple for procedure simplicity, and safe and more safe for reagents safety. Each attribute was given a weightage as an indication of its importance in DNA extraction method selection and the sum of all weightage is equal to 1. The SAW technique was performed using the following equation [28]:

$$S_{i}=\overset{6}{\underset{j=1}{\sum }}w_{j}r_{ij\;}\;for\;i=1,2,3,4,5$$

where *S_i_* is overall score, *w_j_* is weightage of jth attribute, and *r_ij_* is standardised value of the *i*th DNA extraction method with respect to the *j*th attribute. For beneficial attribute, *r_ij_* = *x_ij_*/*x_ij_*_(max)_ and for non-beneficial attribute, *r_ij_* = *x_ij_*_(max)_/*x_ij_*_,_ where *x_ij_* is original value and *x_ij_*_(max)_ is the largest value of the *j*th attribute of the *i*th DNA extraction method [29,30]. DNA extraction method with the highest overall score was granted the highest ranking, hence identified as the optimal extraction method.

## 3. Results

### 3.1. DNA Concentration

Table 1 shows DNA concentration of 13 EBN samples extracted using five different DNA extraction methods and measured using spectrophotometric assay. Regardless of extraction methods used, the processed EBNs (samples 12–13) which have undergone intensive degree of processing generally have shown lower DNA concentration than the unprocessed EBNs samples (samples 1–11). Among the three commercial kits tested, the Wizard and Qiagen methods gave significantly highest DNA concentration (*p* < 0.05) of 2.23–5.03 ng/µL and 1.35–4.50 ng/µL, respectively. Interestingly, in Qiagen method, four EBNs (samples 8–11) which originated from *A. maximus* produced significantly lower amount of extracted DNA than others (*p* < 0.05). NucleoSpin method yielded significantly lowest DNA concentration for EBN samples (*p* < 0.05) ranging from 0.30 to 1.25 ng/µL. The SDS method, which is a standard method and widely used in DNA extraction of EBN [4,31] however, gave relatively low DNA concentration in this study. Despite its low amount of extracted DNA, the SDS method showed significantly greater ability in extracting DNA from *A. maximus* EBNs (samples 8–11) than *A. fuciphagus* EBNs (*p* < 0.05). The hybrid SDS/Qiagen method showed a significant improvement in DNA recovery compared to the SDS method with at least 2-fold’s increment. It yielded significantly highest DNA concentration for EBN samples (*p* < 0.05) ranging from 4.18 to 5.68 ng/µL.

**Figure 1:**
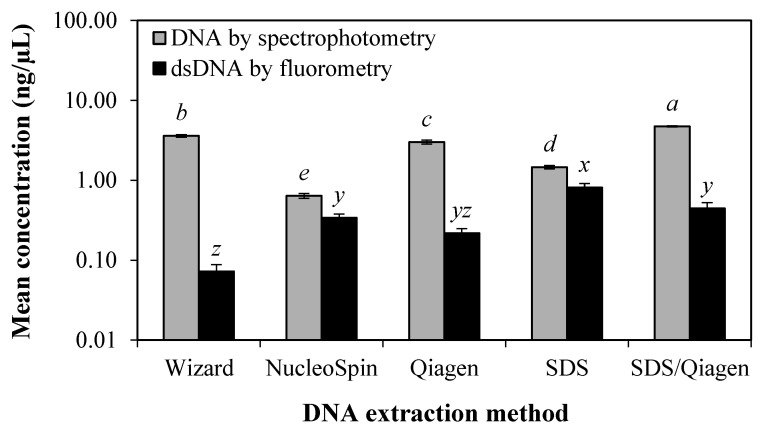
Mean DNA and dsDNA concentrations of EBN samples extracted with five different DNA extraction methods as measured by spectrophotometry and fluorometry, respectively. Different letters in each quantification method indicate significant differences (*p* < 0.05). Values are mean ± standard error with samples size *n* = 52 (spectrophotometry) and *n* = 13 (fluorometry).

Comparing quantification assays, the average DNA and dsDNA concentrations of all EBN samples from five different DNA extraction methods quantified by spectrophotometry and fluorometry, respectively, are shown in Figure 1. The average DNA concentration via SDS/Qiagen method was significantly highest (*p* < 0.05) at 4.73 ng/µL by absolute value, followed by Wizard, Qiagen, SDS and NucleoSpin methods using the spectrophotometric quantification. For fluorometric quantification, the SDS method gave the highest dsDNA concentration while the Wizard method yielded the lowest for all EBN samples. 

**Table 1:** DNA concentration of EBN samples extracted with five DNA extraction methods as measured by spectrophotometry.

EBN	Description			DNA Concentration (ng/μL) ^†^				
	Type	Species ^‡^	Origin	Wizard	NucleoSpin	Qiagen	SDS	SDS/Qiagen
1	Unprocessed	*A. fuciphagus*	* Segamat, Johor	3.65 ± 0.21 ^b^	1.25 ± 0.35 ^c^	4.33 ± 0.33 ^ab^	1.10 ± 0.18 ^c^	4.60 ± 0.42 ^a^
2	Unprocessed	*A. fuciphagus*	* Kapar, Selangor	4.40 ± 0.54 ^a^	0.83 ± 0.05 ^b^	3.65 ± 0.61 ^a^	1.58 ± 0.15 ^b^	4.23 ± 0.72 ^a^
3	Unprocessed	*A. fuciphagus*	* Nibong Tebal, Penang	3.33 ± 0.13 ^ab^	0.45 ± 0.13 ^c^	3.23 ± 0.90 ^b^	1.35 ± 0.06 ^c^	4.43 ± 0.73 ^a^
4	Unprocessed	*A. fuciphagus*	* Klang, Selangor	3.55 ± 0.13 ^c^	0.60 ± 0.08 ^e^	4.08 ± 0.25 ^b^	1.35 ± 0.13 ^d^	4.78 ± 0.21 ^a^
5	Unprocessed	*A. fuciphagus*	** Sarikei, Sarawak	3.80 ± 0.81 ^a^	0.60 ± 0.08 ^b^	4.50 ± 0.26 ^a^	1.10 ± 0.08 ^b^	4.45 ± 0.19 ^a^
6	Unprocessed	*A. fuciphagus*	** Gomantong Cave, Sabah	2.23 ± 0.43 ^c^	0.50 ± 0.08 ^d^	3.75 ± 0.53 ^b^	0.70 ± 0.00 ^d^	4.98 ± 0.44 ^a^
7	Unprocessed	*A. fuciphagus*	** Baram, Sarawak	5.03 ± 0.78 ^a^	1.23 ± 0.15 ^c^	3.38 ± 0.10 ^b^	1.83 ± 0.10 ^c^	4.65 ± 0.70 ^a^
8	Unprocessed	*A. maximus*	** Gomantong Cave, Sabah	3.20 ± 0.87 ^b^	0.45 ± 0.13 ^d^	1.35 ± 0.26 ^cd^	1.65 ± 0.19 ^c^	5.18 ± 0.68 ^a^
9	Unprocessed	*A. maximus*	** Niah Cave, Sarawak	3.30 ± 0.70 ^b^	0.68 ± 0.10 ^d^	1.55 ± 0.13 ^cd^	1.73 ± 0.15 ^c^	5.10 ± 0.75 ^a^
10	Unprocessed	*A. maximus*	** Niah Cave, Sarawak	4.30 ± 0.61 ^a^	0.35 ± 0.06 ^c^	1.90 ± 0.46 ^b^	2.28 ± 0.56 ^b^	5.68 ± 1.08 ^a^
11	Unprocessed	*A. maximus*	** Subis Cave, Sarawak	4.50 ± 0.60 ^a^	0.55 ± 0.06 ^c^	1.58 ± 0.22 ^b^	2.15 ± 0.21 ^b^	4.75 ± 0.49 ^a^
12	Processed	*A. fuciphagus*		2.55 ± 0.37 ^b^	0.53 ± 0.10 ^c^	4.28 ± 0.33 ^a^	0.73 ± 0.15 ^c^	4.18 ± 0.50 ^a^
13	Processed	*A. fuciphagus*		2.95 ± 0.13 ^b^	0.30 ± 0.00 ^d^	1.53 ± 0.13 ^c^	1.40 ± 0.14 ^c^	4.50 ± 0.16 ^a^
			Average	3.59 ± 0.49	0.64 ± 0.11	3.01 ± 0.35	1.46 ± 0.16	4.73 ± 0.54

^‡^ *A. fuciphagus*, *Aerodramus fuciphagus*; *A. maximus*, *Aerodramus maximus*. ^†^ Values are mean ± standard deviation with *n* = 4 and different superscript letters in the same row indicate significantly different (*p* < 0.05). * Peninsular Malaysia; ** East Malaysia.

### 3.2. DNA Purity

**Figure 2:**
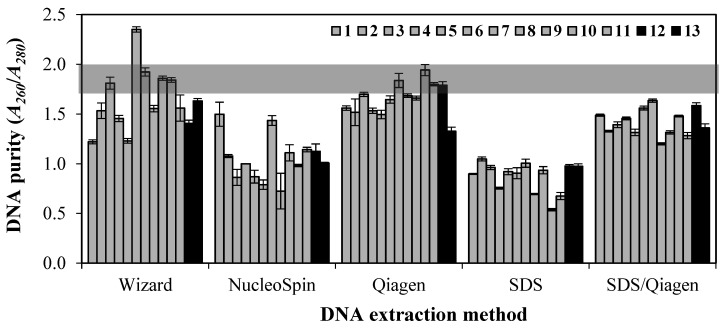
Comparison of purity of DNA extracted from 13 EBN samples with five different DNA extraction methods. Samples 1–11 are unprocessed EBNs and samples 12–13 are processed EBNs. Grey shaded area represents satisfactory range for pure DNA from 1.7 to 2.0. Values are mean ± standard error with samples size.

Figure 2 shows the purity of extracted DNA from EBN samples determined by the absorbance ratio of 260 and 280 nm (A_260_/A_280_) where A_260_ and A_280_ values indicated the presence of DNA and protein, respectively. The extracted DNA is considered pure if A_260_/A_280_ value ranged between 1.7 and 2.0 [32]. The Wizard and Qiagen methods obtained the highest DNA purity. A closer observation showed that Qiagen method had a higher sampling fraction of 6/13 than the Wizard method with 4 samples out of 13. Contrarily, the NucleoSpin, SDS and SDS/Qiagen methods gave relatively low DNA purity ranging from 0.87 to 1.42. Figure 2 also shows that the purity of extracted DNA was not significantly different between processed and unprocessed EBNs in any extraction method suggesting processing of EBN does not affect DNA purity.

### 3.3. PCR Amplifiability

**Figure 3:**
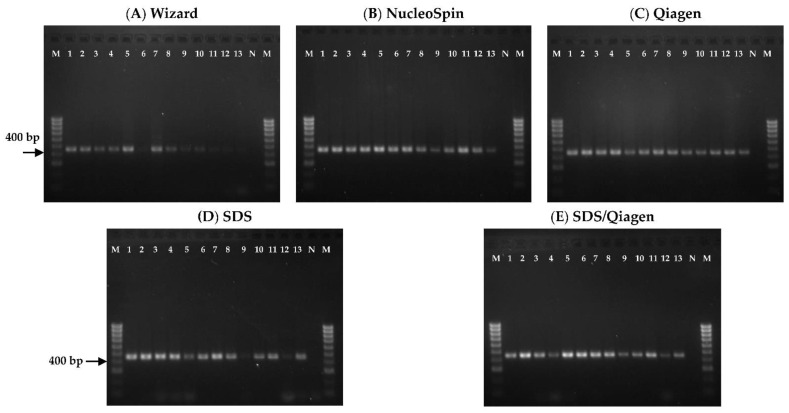
Gel electrophoreses of the 406 bp PCR products of cytochrome b gene amplified from extracted DNA of EBN samples with five different DNA extraction methods, namely (**A**) Wizard method, (**B**) NucleoSpin method, (**C**) Qiagen method, (**D**) SDS method and (**E**) hybrid SDS/Qiagen method. Lane M, 100 bp DNA ladder; lane 1–11, unprocessed EBNs; lane 12–13, processed EBNs; lane N, no template control.

Figure 3 shows PCR amplification results using a pair of cytochrome b gene primers at expected size of 406 bp. The extracted DNA of unprocessed EBNs was successfully amplified while the processed ones (lanes 12–13) showed relatively faint PCR bands. The Wizard method gave no visible lanes 12 and 13. Weak PCR bands appeared in the NucleoSpin (lane 13) and SDS (lane 12) while Qiagen and SDS/Qiagen gave reasonable PCR bands for lanes 12 and 13. From the five DNA extraction methods, only DNA extracted with Qiagen method gave consistently intense PCR bands with expected size for all EBN samples.

### 3.4. Time, Safety, and Economic Evaluation of Extraction Methods

Based on a single sample handling [33], commercial kits required less time for DNA extraction than the SDS and SDS/Qiagen methods (Table 2). From the five DNA extraction methods, the commercial kits employed less hazardous reagents than SDS and SDS/Qiagen methods which required the use of corrosive and flammable reagents such as SDS, CTAB, chloroform/isoamyl alcohol and isopropanol. Most of the reagents used in all five DNA extraction methods were classified as skin and eyes irritant, and they were less likely to cause harmful effects if handled with care [34]. The most economical DNA extraction method was the SDS method, followed by SDS/Qiagen method. The reagents used in SDS method were common and often purchased in bulk quantity, thus it is cheapest in extraction cost. The three commercial kits were the most expensive. Comparing between the commercial kits, the Qiagen method had the lowest extraction cost with estimation of USD 3.00 for one sample, followed by Wizard and NucleoSpin methods at USD 3.40 and USD 4.00, respectively. The cost for a single sample DNA extraction has been estimated based on average reagents and commercial kits prices in Malaysia. The cost attribute was not included in this DNA extraction method selection due to subjectivity of reagent costs for the SDS and SDS/Qiagen methods.

### 3.5. Optimal DNA Extraction Method with SAW Technique

Table 2 shows that Qiagen method ranked first, followed by NucleoSpin, Wizard, SDS and SDS/Qiagen methods. The Qiagen method was identified as the most efficient and feasible DNA extraction method for EBN, yielding the highest success rate of PCR amplification with intense bands and excellent DNA purity, highest procedure simplicity and reagents safety, and required least handling time for DNA extraction. The most widely used conventional and standard method, the SDS was ranked the fourth. Despite obtaining highest amount of DNA, the SDS method gave the lowest DNA purity with relatively lengthy and tedious extraction procedure, and it also involved hazardous reagents. 

### 3.6. Validation of Optimised Qiagen Method for Species Identification of EBN

The Qiagen method was validated to ensure its extracted DNA has the quality for downstream molecular applications. Table 3 shows that all 13 PCR products of the 406 bp cytochrome b gene sequences from EBN samples that were sequenced and subjected to BLASTN homology search were 100% identical to their respective published swiftlet sequences obtained from GenBank database. All the sequences of EBN samples were aligned to their respective swiftlet species sequences, *A. fuicphagus* or *A. maximus* available in GenBank database. These matching obtained BLASTN hits of 100% identity and E-values (Expected values) of 0 indicating that the hits were significantly matched. 

## 4. Discussion

The lower DNA concentration of processed EBNs may be related to DNA deterioration during processing which typically involves overnight soaking and drying of EBN [4]. It was evident that thermal processing of drying, cooking, baking, and roasting can cause DNA degradation in foods [23]. This trend was consistent with the findings published by Pirondini et al. [33] and Besbes et al. [35], who have reported higher amount of DNA in fresh milk and seafood than in their processed products. The DNA concentration was consistently higher than the dsDNA concentration, regardless of samples and extraction methods used (Figure 1). This could be due to the overestimation of DNA concentration by spectrophotometry as UV absorbance measurement are not selective and cannot distinguish DNA, RNA, or protein [36,37]. The fluorometric assay is also known to be more sensitive and specific for dsDNA only via fluorescent dyes binding, and it minimises the interference of RNA, protein and aromatic compounds in the extracted DNA [38]. As the fluorometric quantification provided a more selective, sensitive, and accurate method for quantifying nucleic acids than the spectrophotometric quantification, the dsDNA concentration was selection for subsequent extraction process. 

In terms of DNA purity, Qiagen method was more superior in removing protein contaminants and inhibitors from EBN when compared with NucleoSpin, SDS, and SDS/Qiagen. This could probably due to protein contamination and organic solvents carryover in the extracted DNA of EBN samples. Generally, protein contamination and residual reagents such as ethanol, phenol, and chloroform interfere the A_260_/A_280_ values and reduce the purity values to below 1.7 [34,39]. The residual reagents contamination may be effectively removed while maintaining the assay sensitivity using commercial nucleic acid extraction kit reagents such as GenElute Maxiprep binding columns [40]. The DNA purity from NucleoSpin and SDS methods may be optimised by adding filtration step with QIAquick spin column from Qiagen kit.

In using SAW technique to select the optimal DNA extraction method for EBN, the multiple contradictory attributes comprising dsDNA concentration, purity, PCR amplifiability, handling time, procedure simplicity, and reagents safety, PCR amplifiability was assigned with a higher weightage than other attributes because a successful PCR amplification is crucial for the subsequent molecular analysis, such as DNA sequencing [41]. It was necessary to consider other attributes of optimum DNA extraction method, such as handling time, procedure simplicity, safety of reagents used [5,42], and costs besides extracted DNA quality and quantity. The expensive cost of commercial kits is related to its sophisticated reagents and columns that are covered by international patents [33]. The handling time is directly proportional to procedure simplicity, where commercial kits contained simply fewer steps in extraction procedure than the SDS and SDS/Qiagen methods have shorter handling time. Most of the reagents needed were readily provided in the commercial kits. DNA extraction techniques employed in commercial kits was simpler than precipitation technique used in conventional SDS method, i.e., silica paramagnetic particles-based technique in Wizard method and column-based technique in NucleoSpin and Qiagen methods. Safety of reagents was evaluated following the material and safety datasheet (MSDS). In brief, commercial kits were fast, simple and safe but expensive whereas conventional methods were slow, tedious, and hazardous, but economical. Hybrid method was safer and faster than conventional methods, and less expensive than commercial kits.

The Qiagen method, found to be the optimal extraction method for EBN in this study, however, contradicts Wu et al. [1] who reported that NucleoSpin method was their best in EBN studies for successful PCR amplification. The difference in findings may be due to the variation in sample, DNA extraction method or the targeted gene of interest used for amplification. This is shown in this study when different extraction methods were suitable for DNA extraction of different species of EBN. The Qiagen method was more suitable for *A. fuciphagus* than *A. maximus* whereas the SDS method showed significantly greater ability in extracting EBN’s DNA from *A. maximus* than *A. fuciphagus*. This may be due to the different nature and composition of food from different species which affected the DNA extraction [34]. The best PCR amplifiability results by Qiagen method may be attributed to the higher quality of DNA extracted. The weak PCR bands could be due to DNA degradation and fragmentation which occurred during EBN processing [4]. Nonetheless, the success rate of PCR amplification was not correlated to the concentration and purity of extracted DNA of EBN. For instance, the Wizard method yielded high amount of DNA with good purity but it gave relatively faint PCR bands for most of the amplified EBN samples. This observation is in agreement with previous work by Turci et al. [38], who reported unsuccessful amplification in most of the tomatoes products although high amounts of extracted DNA was yielded. 

## 5. Conclusions

The SAW analysis has helped in determining optimal DNA extraction method for EBN species identification through end-point PCR. The hybrid DNA extraction method (SDS/Qiagen) was developed by replacing the DNA precipitation step with QIAquick spin column from the Qiagen method to improve DNA recovery of the SDS method which has shown great improvement as the silica-based column has greater DNA binding ability in the presence of chaotropic salts more efficiently. The hybrid method provides an alternative for a lower cost method than the commercial kits while being more rapid when compared to the conventional method and without compromise of accuracy. The extracted DNA recovery, purity and PCR amplifiability has improved over the conventional method thus can also be recommended as an efficient and feasible method for a more sustainable or routine analysis for EBN identification. With no consideration on cost, the commercial kit, Qiagen method ranked the best in terms of highest DNA purity and PCR amplifiability for DNA sequencing to identify swiftlet species of EBN.

## Figures and Tables

**Table 2:** Evaluation of five different DNA extraction methods using simple additive weighting technique.

DNA Extraction Method	Attribute/Measured Data						Overall Score	Rank
	dsDNA ^‡^	Purity ^†^	PCR ^†^	Time ^‡^	Simplicity ^Φ^	Safety ^Φ^		
Weightage (Σ = 1)	1/6	1/6	2/6	1/6	1/6	1/6		
Wizard	0.08	0.31	0.54	2.0	2	2	1.02	3
NucleoSpin	0.34	0.00	0.85	2.0	2	2	1.06	2
Qiagen	0.22	0.46	1.00	2.0	2	2	1.25	1
SDS	0.81	0.00	0.85	4.5	1	1	0.78	4
SDS/Qiagen	0.44	0.00	0.92	4.0	1	1	0.75	5

^‡^ dsDNA concentration measured by fluorometry (ng/mL); handling time (hours). ^†^ Sampling fraction with purity between 1.7 and 2.0 or successful PCR amplification with intense bands, respectively. ^Φ^ Direct rating of procedure simplicity, 1, simple; 2, more simple, and reagents safety, 1, safe; 2, more safe.

**Table 3:** BLAST results on GenBank with first hit sequence using 406 bp of cytochrome b gene marker.

EBN	First Hit Sequence (Species and Accession Number)	Maximum Identity (%)	E-Value ^†^
1	*Aerodramus fuciphagus* (JQ353840.1)	100%	1 × 10^−87^
2	*Aerodramus fuciphagus* (JQ353840.1)	100%	1 × 10^−87^
3	*Aerodramus fuciphagus* (JQ353840.1)	100%	1 × 10^−87^
4	*Aerodramus fuciphagus* (JQ353840.1)	100%	1 × 10^−87^
5	*Aerodramus fuciphagus* (JQ353840.1)	100%	1 × 10^−87^
6	*Aerodramus fuciphagus* (JQ353840.1)	100%	1 × 10^−87^
7	*Aerodramus fuciphagus* (JQ353840.1)	100%	1 × 10^−87^
8	*Aerodramus maximus* (JQ353847.1)	100%	0.0
9	*Aerodramus maximus* (JQ353847.1)	100%	0.0
10	*Aerodramus maximus* (JQ353847.1)	100%	0.0
11	*Aerodramus maximus* (JQ353847.1)	100%	0.0
12	*Aerodramus fuciphagus* (JQ353840.1)	100%	1 × 10^−87^
13	*Aerodramus fuciphagus* (JQ353840.1)	100%	1 × 10^−87^

^†^ E-value the number of hits one can “expect” to see by chance when searching a database of a particular size on BLAST search.
